# The mediating role of COVID-19 anxiety on the relationship between quality of life and spiritual well-being, and hopelessness: A study on cancer patients

**DOI:** 10.1017/S1478951524001238

**Published:** 2024-11-07

**Authors:** Ebru Dığrak, Irfan Akkoç

**Affiliations:** Faculty of Health Sciences, Department of Nursing, Izmir University of Economics, Izmir, Turkiye

**Keywords:** Cancer, COVID-19, hope, spirituality, quality of life

## Abstract

**Objectives:**

The coronavirus pandemic has caused concern in the community, especially in patients. Spirituality, hopelessness, and quality of life have an impact on the management of the process in cancer patients during these crisis periods. To investigate COVID-19 anxiety’s mediating role in hopelessness’ relationships with the quality of life and spiritual well-being among cancer patients.

**Methods:**

This study used a cross-sectional design to collect data from cancer patients using self-administered questionnaires. The study recruited 176 cancer patients receiving treatment at a university hospital. The participants completed measures of spiritual well-being, COVID-19 anxiety, hopelessness, and quality of life. Following preliminary analyses, a mediation model was analyzed using the PROCESS macro for SPSS, with the bootstrap method applied (model 4).

**Results:**

The results showed that spiritual well-being was negatively associated with COVID-19 anxiety and hopelessness, and positively associated with the quality of life. COVID-19 anxiety was associated positively with hopelessness, and negatively with the quality of life. Moreover, COVID-19 anxiety mediated the relationship between hopelessness, spiritual well-being, and quality of life.

**Significance of results:**

This study provides evidence for COVID-19 anxiety’s mediating role in the relationship between spiritual well-being and quality of life and hopelessness among cancer patients. The findings suggest that interventions aimed at reducing COVID-19 anxiety may be effective in reducing hopelessness among cancer patients, by promoting higher levels of spiritual well-being and improving quality of life.

## Introduction

Cancer is an important societal, public health, and economic problem worldwide. According to the findings, an estimated 20 million cancer cases were newly diagnosed in 2022 and 9.7 million people died from the disease worldwide. By 2050, the number of cancer cases is predicted to reach 35 million (Bray et al. 2024). Cancer profoundly affects individuals not only through the direct consequences of getting cancer, but also through physical, psychological, and social problems for patients and survivors (Wang and Feng 2022). In addition, these physical, psychological, and social problems experienced by many cancer patients after diagnosis and during the treatment process lead to hopelessness (Nierop-van Baalen et al. 2020).

Hopelessness consists of emotional, cognitive, and motivational components characterized by negative expectations about the future (Saricali et al. 2022). Hopelessness is a significant concern for cancer patients from the moment of diagnosis, and the extended duration of the disease, complex treatments, uncertainty of recurrence, and associated costs can exacerbate this feeling (Madani et al. 2018). Cancer patients, especially those in advanced stages of cancer, are more prone to hopelessness, which can jeopardize the patient’s physical and mental health (Nierop-van Baalen et al. 2020; Rawas et al. 2024). In addition, while cancer is a challenging process that affects the individual with its treatment and life in general, the COVID-19 pandemic has affected this process and caused anxiety in cancer patients. Especially during the pandemic period, it was observed that individuals with moderate or poor general health status had higher anxiety symptoms in cancer patients (Adzrago et al. 2022).

COVID-19 anxiety refers to dysfunctional anxiety associated with the COVID-19 pandemic. Encouraging cancer patients to stay at home during the pandemic has led to disruption of their treatment, disease progression, and economic crisis, and the negative effects of these crises have caused COVID-19 anxiety (Ciążyńska et al. 2020). A study showed that COVID-19 anxiety was associated with increased hopelessness in cancer patients during the pandemic (Büyükbayram et al. 2022). In addition, COVID-19 anxiety and hopelessness experienced by patients negatively affect the treatment and spiritual well-being of cancer patients (Eskelinen et al. 2014; Luo et al. 2024).

Spiritual well-being refers to nonphysical ways of finding health and/or inner peace, such as meditating, seeking meaning and purpose in life (Jimenez-Fonseca et al. 2018). By increasing positive coping attitudes, spiritual well-being can facilitate cancer patients’ adaptation to the disease and improve their coping skills in the face of difficulties (Vincensi 2019). Cancer patients have been found to show a high level of interest in spiritual well-being (Al-Natour et al. 2017) and it has been suggested that spiritual well-being protects them against hopelessness (Saarelainen 2019; Gheihman et al. 2016). Therefore, spirituality is an important factor for COVID-19 anxiety and hopelessness in cancer patients. Based on these relationships, COVID-19 anxiety is expected to mediate the relationship between spiritual well-being and hopelessness and Hypothesis 1 was formed.

Hypothesis 1: COVID-19 Anxiety mediates the relationship between Spiritual Well-Being and Hopelessness.

In recent years, because of oncology practices, new drugs, and therapeutic approaches, cancer has become not only an acute disease but also a chronic disease. Therefore, cancer patients are living longer but struggle with the long-term consequences of cancer and its treatment, which affects quality of life (Arndt et al. 2017; Firkins et al. 2020; Thong et al. 2019). Quality of life is a multidimensional construct that encompasses perceptions of both positive and negative dimensions such as physical, emotional, social, and cognitive functioning (Nayeri et al. 2020). Quality of life is lower in cancer patients than in the general population (Flyum et al. 2021). Even in a meta-analysis study, it is known that even in cancer survivors, physical and mental quality of life is low (Firkins et al. 2020). Feelings of uncertainty, anxiety, and hopelessness caused by cancer also affect quality of life and cause a decrease in quality of life (Ravindran et al. 2019). Although there is no study on cancer patients, according to a meta-analysis study conducted during the COVID-19 pandemic, it was reported that anxiety and hopelessness increased and quality of life decreased in individuals (Pappa et al. 2020). Based on these relationships, COVID-19 anxiety is expected to mediate the relationship between quality of life and hopelessness, and Hypothesis 2 was formed.

Hypothesis 2: COVID-19 Anxiety mediates the relationship between Quality of Life and Hopelessness.

**Figure 1.**
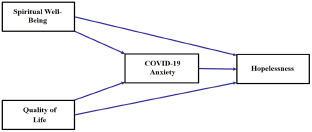
Study concept model.

Quality of life, spiritual well-being, hopelessness, and COVID-19 anxiety are important factors for cancer patients, but to the best of our knowledge, only direct links have been examined so far. No studies focusing on these 4 variables were found in the literature review. To improve our understanding of these relationships, we began to investigate the mechanisms underlying the links between quality of life, spiritual well-being, hopelessness, and COVID-19 anxiety in cancer patients. Based on the above, it is expected that (1) COVID-19 anxiety mediates the relationship between quality of life and spirituality and (2) quality of life and hopelessness (Fig. 1). It is thought that this study will provide important implications for the physical, psychological, and social problems of cancer patients and contribute to the literature.

## Methods

### Study design and sampling method

The population of the study was an education-research hospital located in western Türkiye. It is the largest private education-research hospital in the west of the country and oncology patients are treated both as outpatients and inpatients. Patients treated in the outpatient chemotherapy unit were included in the study. The outpatient chemotherapy unit was preferred because of the ease of access to the patients and the suitable environment and sufficient time for comfortable interviews. The sample size was calculated as 152 individuals considering a margin of error of 5% within a 95% confidence interval (Sekaran 2019). A total of 176 patients who met the inclusion criteria were included in the study in the study between August 20 and October 15, 2022. Inclusion criteria were (1) patients who were take chemotherapy in the outpatient chemotherapy units; (2) age 18 years and older; and (3) being able to read and write in Turkish. The participants voluntarily agreed to participate and all participating cancer patients provided informed oral consent prior to completing the questionnaire.

### Data collection forms

#### Patient information form

This form consists of 2 sections personel characteristics (age, gender, education level, marital status, family type, employment status) and disease characteristics (cancer type, diagnosis time, treatment time).

#### Coronavirus Anxiety Scale

This is a 1-dimensional 5-item scale developed by Lee (2020) to measure of participants’ coronavirus anxiety. The Cronbach’s alpha reliability coefficient of the original scale was reported as 0.93. The validity and reliability of the Turkish version of the scale were conducted by Akkuzu et al. (2020), and its Cronbach alpha reliability coefficient was found to be 0.81. The scale obtained answers with a 5-point likert scale (1 = strongly disagree; 5 = strongly agree).

#### Beck Hopelessness Scale

The scale was developed by Beck et al. (1974) to measure the cognitive component of depression. The validity and reliability study of the Turkish language version was carried out by Durak and Palabıyıkoğlu in 1994 and Cronbach’s alpha internal reliability coefficient of the scale was found 0.86. The scale consists of 20 questions and focuses on 3 important aspects: loss of motivation, feelings about the future, and expectations.

#### Spiritual Well-Being Scale *(FACİT-SP-12)*

It was developed by Peterman et al. (2002) to assess the mental well-being of cancer patients and individuals with chronic diseases. The Cronbach alpha reliability coefficient of the original scale was between 0.81 and 0.83. In the study conducted by Aktürk et al. (2017) on the validity and reliability of the Turkish version of the scale, the Cronbach alpha reliability coefficient of the scale was found between 0.78 and 0.93. It is a 5-point likert-type (1 = strongly disagree; 5 = strongly agree) scale consisting of 12 items. The scale has 3 subdimensions: meaning, peace, and faith. The total score of the scale varies between 0 and 48 and a high score indicates a high level of spiritual well-being.

#### Quality of Life Scale (EORTC QLQ-C30)

The questionnaire was developed by the European Organization for Research and Treatment of Cancer Quality of Life Questionnaire to measure the physical, psychological, and social functions of cancer patients (Fayers et al. 2001). The scale consists of 30 items, 5-point likert type (1 = strongly disagree; 5 = strongly agree) and 3 subdimensions (global health status, functional, symptom). The validity and reliability study of the Turkish version was conducted by Cankurtaran et al. in (2008) and the Cronbach alpha internal reliability coefficient of the scale was found to be 0.70.

#### Statistical analysis

Data analysis was conducted using SPSS, with a level of statistical significance set at a 95% confidence interval. The reliability of the scales was assessed by calculating Cronbach’s alpha reliability coefficient. Descriptive statistics were used to describe sociodemographic variables. Pearson correlation analysis was used to examine the relationships between study variables. Following preliminary analyses, a mediation model was analyzed using the PROCESS macro for SPSS, with the bootstrap method applied.

We employ the Bootstrap method to scrutinize the mediation effects encapsulated in Model 4, which comprises mediation relationships. To analyze the total, direct, and indirect effects of the variables within this study model, we utilize the Bootstrap method, incorporating 5000 resamplings, a 95% symmetric confidence interval, and a 95% confidence interval rectified for bias. Our investigation exploits the Bootstrap method to ascertain the significance of the mediating role of COVID-19 Anxiety, the mediating variable in this context, in the linkage between the independent and dependent variables. The mediating role is deemed significant if neither the bootstrap upper (BootULCI) nor lower (BootLLCI) confidence interval limits at the 95% level contain 0 (Hayes 2018).

## Results

### Participants’ characteristics

Among the cancer patients included in the study, 59.1% were female, 46.6% were primary school graduates, 78.4% were married, 58% were not working, and 75% lived with their nuclear family. The average age of the cancer patients were 58.05 ± 13.27 and ranging from 26 to 88. The most prevalent cancer type were lung cancer (22.7%) and breast cancer (13.6%); 61.4% were not metastasis; 51.1% received a diagnosis in the prior 6 months, and 60.2% were presence of another chronic disease Table 1.

**Table 1.** Demographic characteristics and medical histories of participants

Variables	Category	Frequency	Percent
Age		*M* = 58.05	SD = 13.27
Gender	Female	104	59.1
	Male	72	40.9
Educational status	Primary school	82	46.6
	High school	42	23.9
	University	52	29.5
Marital status	Married	138	78.4
	Single	38	21.6
Family type	Nuclear family	132	75.0
	Extended family	44	25.0
Employment status	Yes	74	42.0
	No	102	58.0
Clinical diagnosis	Lung CA	40	22.7
	Breast CA	24	13.6
	Colon CA	16	9.1
	Ovarian CA	16	9.1
	Cervix CA	8	4.5
	Gastric CA	6	3.4
	Another cancers*	66	37.6
Metastasis	Yes	68	38.6
	No	108	61.4
Duration of treatment	6 months ago	90	51.1
	6−12 months ago	36	20.5
	1−2 years ago	34	19.3
	2−3 years ago	16	9.1
Presence of another chronic disease	Yes	106	60.2
	No	70	39.8

* *Note*: Pancreatic CA, Prostate CA, Liver CA, Rectum CA, Testis CA, Skin CA, Gallbladder CA.

### Correlations

Table 2 shows the findings regarding the reliability, mean, standard deviation, and correlation analysis of the data obtained from cancer patients regarding the variables of COVID-19 anxiety, spiritual well-being, hopelessness, and quality of life. The research model found significant relationships between dependent, independent, and mediator variables. The results of the correlation analysis reveal positive correlation versus negative correlation between the independent variable (spiritual well-being and quality of life) and the dependent variable (hopelessness), and the mediator variable (COVID-19 anxiety).

**Table 2.** Reliability, mean, standard deviation, and correlation values

	M	SD	1	2	3	4	5	6	7	8	9	10
1. Meaning	3.96	0.88	**(.86)**									
2. Peace	3.41	0.94	.74**	**(.75)**								
3. Faith	3.45	1.09	.22**	.10	**(.78)**							
4. Hope	1.21	0.29	−.60**	−.59**	−.34**	**(.82)**						
5. Loss of Motivation	1.26	0.30	−.62**	−.55**	−.23**	.69**	**(.80)**					
6.Expectations Toward the Future	1.32	0.28	−.69**	−.62**	−.24**	.78**	.72**	**(.72)**				
7. Functional	2.99	0.63	.42**	.45**	.27**	−.43**	−.38**	−.43**	**(.92)**			
8. Symptom	2.11	0.66	−.37**	−.39**	−.21**	.33**	.31**	.34**	−.91**	**(.91)**		
9. Global Health Status	2.83	1.23	.20**	.25**	−.08	−.19*	−.20**	−.28**	.09	−.12	**(.90)**	
10.COVİD-19 Anxiety	1.23	0.51	−.42**	−.30**	−.20**	.21**	.39**	.28**	−.21**	.19*	−.17*	**(.94)**

** *Note*: *p* ≤ .01, Alpha reliability coefficients are shown in parentheses.

Tables 3 and 4 illustrate the total, direct, and indirect effects, as well as the path coefficients of the independent variable’s analysis on each dependent variable within the research model. These tables offer insights into how the independent variables impact the dependent variables, as well as the role of COVID-19 Anxiety as a mediating variable within these relationships.

**Table 3.** Total, direct, and indirect effects in the model

		Indirect			Direct			Total		
Outcome	Predictors	ß	BootLLCI	BootULCI	ß	LLCI	ULCI	ß	LLCI	ULCI
Hope	Faith	−.03	−.0841	.0026	−.31***	−.1225	−.0461	−.34***	−.1298	−.0543
Loss of motivation	Faith	−.07	−.1466	−.0173	−.16*	−.0825	−.0058	−.23***	−.1044	−.0238
Expectations toward the future	Faith	−.05	−.1028	−.0108	−.19**	−.0884	−.0133	−.24**	−.1010	−.0254
Hope	Peace	−.01	−.0785	.0501	−.58***	−.2205	−.1417	−.59***	−.2218	−.1467
Loss of motivation	Peace	−.07	−.1443	−.0264	−.47***	−.1919	−.1111	−.54***	−.2159	−.1356
Expectations toward the future	Peace	−.03	−.0767	.0042	−.59***	−.2144	−.1403	−.62***	−.2220	−.1510
Hope	Meaning	.02	−.0640	.0983	−.62***	−.2515	−.1636	−.60***	−.2403	−.1604
Loss of motivation	Meaning	−.07	−.1481	−.0072	−.54***	−.2315	−.1436	−.61***	−.2520	−.1709
Expectations toward the future	Meaning	.00	−.0390	.0519	−.69***	−.2634	−.1860	−.69***	−.2581	−.1879

*Note*: BootLLCI = lower limit of the bootstrap confidence interval with %95; BootULCI = upper limit of the bootstrap confidence interval with 95%; bootstrap sampling size = 5000;

*** *p* < .001;

** *p* < .01;

* *p* < .05.

**Table 4.** Total, direct, and indirect effects in the model

		Indirect			Direct			Total		
Outcome	Predictors	ß	BootLLCI	BootULCI	ß	LLCI	ULCI	ß	LLCI	ULCI
Hope	Functional	−.03	−.0783	.0050	−.40***	−.2512	−.1242	−.43 ***	−.2621	−.1371
Loss of motivation	Functional	−.07	−.1359	−.0130	−.31***	−.2098	−.0826	−.38 ***	−.2451	−.1127
Expectations toward the future	Functional	−.04	−.0870	−.0080	−.39***	−.2348	−.1132	−.43 ***	−.2530	−.1316
Hope	Symptom	.03	−.0011	.0851	.30***	.0726	.1998	.33 ***	.0856	.2118
Loss of motivation	Symptom	.07	.0131	.1293	.24***	.0497	.1746	.31 ***	.0764	.2076
Expectations toward the future	Symptom	.04	.0078	.0882	.30***	.0671	.1884	.34 ***	.0845	.2067
Hope	Global Health Status	−.03	−.0763	.0170	−.16*	.0294	.0983	−.19 ***	−.0791	−.0096
Loss of motivation	Global Health Status	−.07	−.1277	.0163	−.13	−.0666	.0009	−.20 ***	−.0843	−.0129
Expectations toward the future	Global Health Status	−.04	−.0882	.0083	−.24***	−.0866	−.0215	−.28 ***	−.0964	−.0305

*Note*: BootLLCI = lower limit of the bootstrap confidence interval with 95%; bootULCI = upper limit of the bootstrap confidence interval with 95%; bootstrap sampling size = 5000;

*** *p* < .001; **p < .01;

* *p* < .05.

### Mediating role of COVID-19 anxiety between the spiritual well-being and hopelessness

With Faith as an independent variable, the indirect effect on Hope is insignificant, as the confidence interval includes 0 (ß = −.03, BootLLCI = −.0841, BootULCI = .0026), but is significant for Loss of Motivation (ß = −.07, BootLLCI = −.1466, BootULCI = −.0173) and expectations toward the future (ß = −.05, BootLLCI = −.1028, BootULCI = −.0108). Direct effects are significant (Hope ß = −.31, *p* < .001; Expectations toward the future ß = −.16, *p* <.01; Loss of Motivation ß = −.19, *p* < .05). The total effects are also significant, indicating a negative relationship between Faith and the outcome variables of Hope (ß = −.34, *p* < .001), Expectations toward the future (ß = −.23, *p* < .001) and Loss of Motivation (ß = −.24, *p* < .01).

Regarding Peace as an independent variable, there is no statistical significance for indirect effects on both Hope (ß = −.01, BootLLCI = −.078, BootULCI = .0501) and Expectations toward the Future (ß = −.03, BootLLCI = −.0767, BootULCI = .0042), as evidenced by a confidence interval that encapsulates 0. However, there is significance for the indirect effect on Loss of Motivation (ß = −.07, BootLLCI = −.1443, BootULCI = −.0264). The direct effects on Hope (ß = −.58), Expectations toward the Future (ß = −.47), and Loss of Motivation (ß = −.59) are statistically significant at level *p* < .001. The total effects also reach statistical significance, further demonstrating a negative relationship between Peace and the outcome variables of Hope (ß = −.59, *p* < .001), Expectations toward the Future (ß = −.54, *p* < .001), and Loss of Motivation (ß = −.62, *p* < .001).

When considering Meaning as an independent variable, there is no statistical significance for indirect effects on both Hope (ß = .02, BootLLCI = −.0640, BootULCI = .0983) and Expectations toward the Future (ß = .00, BootLLCI = −.0390, BootULCI = .0519), as indicated by confidence intervals encompassing zero. However, there is significance for indirect effect on Loss of Motivation (ß = −.07, BootLLCI = −.1481, BootULCI = −.0072). The direct effects on Hope (ß = −.62), Expectations toward the Future (ß = −.54), and Loss of Motivation (ß = −.69) are statistically significant at level *p* < .001. The total effects also reach statistical significance, further demonstrating a negative relationship between Meaning and the outcome variables of Hope (ß = −.60, *p* < .001), Expectations toward the Future (ß = −.61, *p* < .001), and Loss of Motivation (ß = −.69, *p* < .001).

**Figure 2.**
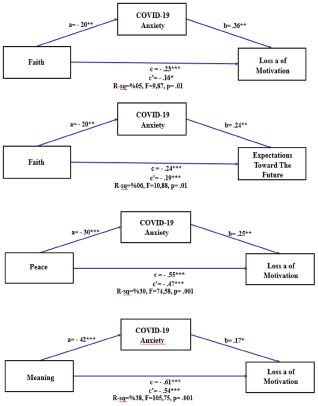
Mediating effects of COVID-19 anxiety on the relationship between spiritual well-being and hopelessness.

The results suggest that COVID-19 Anxiety mediates some relationships between predictors and outcomes (Faith–Loss of motivation; Faith–Expectations toward the future; Peace–Loss of motivation and Meaning–Loss of motivation), and these predictors (Faith, Peace, and Meaning) have significant direct impacts on Hope, Loss of Motivation, and Expectations toward the future. Based on these findings, it can be concluded that mediation Hypothesis 1 is partially supported (Fig. 2).

### Mediating role of COVID-19 anxiety between the quality of life and hopelessness

Table 4 shows the path analysis showing the path coefficients of the relationships between the variables in the model. With Functional as an independent variable, the indirect effects are not statistically significant for Hope (ß = −.03, BootLLCI = −.0783, BootULCI = .0050) as the confidence interval included 0. However, it is statistically significant for both Loss of Motivation (ß = −.07, BootLLCI = −.1359, BootULCI = −.0130) and Expectations toward the Future (ß = −.04, BootLLCI = −.0870, BootULCI = −.0080). The direct effects were significant for all 3 outcomes: Hope (ß = −.40, *p* < .001), Loss of Motivation (ß = −.31, *p* < .001), and Expectations toward the Future (ß = −.39, *p* < .001). The total effects are also significant and negative for all 3 outcome variables (Hope ß = −.43, *p* < .001; Loss of Motivation ß = −.38, *p* < .001; and Expectations toward the Future (ß = −.43, *p* < .001).

Regarding Symptoms as an independent variable, there is no significance for the indirect effect on Hope (ß = .03, BootLLCI = −.0011, BootULCI = .0851) In contrast, there is significance for Loss of Motivation (ß = .07, BootLLCI = .0131, BootULCI = .1293) and Expectations toward the Future (ß = .04, BootLLCI = .0078, BootULCI = .0882). The direct effects are all significant and positive (Hope ß = .30, *p* < .001; Expectations toward the Future ß = .30, *p* < .001; Loss of Motivation ß = .24, *p* < .001). The total effects are also significant and positive for all 3 outcome variables (Hope ß = .33, *p* < .001; Loss of Motivation ß = .31, *p* < .001; and Expectations toward the Future (ß = .34, *p* < .001).

When considering Global Health Status as an independent variable, there is no significance for the indirect effects on Hope (ß = −.03, BootLLCI = −.0763, BootULCI = .0170), Loss of Motivation (ß = −.07, BootLLCI = −.1277, BootULCI = .0163), and Expectations toward the Future (ß = −.04, BootLLCI = −.0882, BootULCI = .0083), as suggested by the confidence intervals encompassing 0. The direct effects are significant for Hope (ß = −.16, *p* < .05) and Expectations toward the Future (ß = −.24, *p* < .001), but not for Loss of Motivation (ß = −.13, *p* > .05). The total effects are all significant and negative (Hope ß = −.19, *p* < .001; Loss of Motivation ß = −.20, *p* < .001; and Expectations toward the Future ß = −.28, *p* < .001).

**Figure 3.**
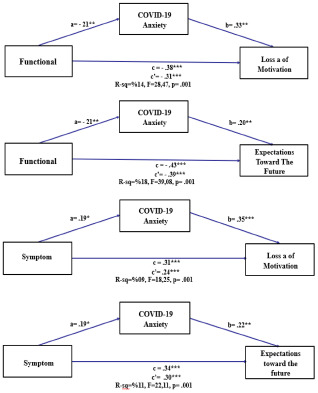
Mediating effects of COVID-19 anxiety on the relationship between quality of life and hopelessness.

From these results, it can be inferred that certain subdimensions of quality of life indirectly affect hopelessness through COVID-19 anxiety (Functional–Loss of motivation; Functional–Expectations toward the future; Symptom–Loss of motivation; and Symptom–Expectations toward the future). Based on these findings, it can be concluded that mediation Hypothesis 2 is partially supported (Fig. 3).

## Discussion

This study aimed to investigate the impact of quality of life and spiritual well-being on the hopelessness of cancer patients, as well as the mediating effect of COVID-19 anxiety on these relationships. The research revealed explanatory findings regarding the relationship among quality of life, hopelessness, spiritual well-being, and COVID-19 anxiety.

According to the findings, there is a partial mediating role of COVID-19 anxiety in the relationship between spiritual well-being and hopelessness among cancer patients and it can be concluded that mediation Hypothesis 1 is partially supported. Studies conducted in Türkiye, where this study was conducted, on diabetes patients, elderly individuals, and individuals diagnosed with COVID-19 during the pandemic have shown negative relationships between spiritual well-being and hopelessness (Buyukbayram et al. 2022; Durmus et al. 2022; Durmus and Ozturk, 2022). Other studies conducted with cancer patients similarly found a significant negative relationship between spiritual well-being and hopelessness (Jimenez-Fonseca et al. 2018; Kirca et al. 2022). During the pandemic, spiritual well-being emerged to escape from hopelessness for patients, and to protect their psychological resilience (Fardin 2020; Khan et al. 2020). During the pandemic period, it was found that cancer patients have a high level of fear of the coronavirus (Erşen et al. 2020), and that there is a positive relationship between hopelessness and COVID-19 anxiety during the pandemic (Kasapoğlu 2022; Lee 2020). Additionally, patients used spiritual well-being to cope with COVID-19 (Hamilton et al. 2022). Kasapoğlu examined COVID-19 anxiety as a mediator variable between spiritual well-being and hopelessness, finding no relationship (Kasapoğlu 2022). No findings have previously been reported regarding the mediating role of COVID-19 anxiety between spiritual well-being and hopelessness specfically among cancer patients. In this study, some dimensions of spiritual well-being indirectly mediated the loss of motivation in some dimensions of hopelessness through COVID-19 anxiety. This result shows that lowered spiritual well-being allows COVID-19 anxiety to increase loss of motivation, and to reduce expectations for the future.

As a result of the analysis, it was found that COVID-19 anxiety partially mediated the relationship between quality of life and hopelessness in cancer patients and it can be concluded that mediation Hypothesis 2 is partially supported. In various studies conducted with cancer patients, a significant negative correlation was found between hopelessness and quality of life (İzci et al. 2018; Ravindran et al. 2019). In a study conducted with women with breast cancer, it was stated that a good quality of life would be achieved when hopelessness was reduced (Pahlevan Sharif et al. 2020). One factor that affects quality of life is anxiety; COVID-19 anxiety has affected individuals’ quality of life (Korkut 2022) and led to lower quality of life (Algahtani et al. 2021; Andrei et al. 2022; Choi et al. 2021; Méndez et al. 2021). This finding is consistent with studies conducted with cancer patients during the pandemic (Ciążyńska et al. 2020). In addition, a positive correlation has been reported between anxiety and hopelessness during the COVID-19 pandemic, and anxiety has been found to increase the level of hopelessness (Andrei et al. 2022; Mert et al. 2022). Anxiety, one of cancer patients’ most common burdens, can be significantly increased by external factors such as COVID-19 (Grajek et al. 2022). In the literature, no previous studies have been found regarding the mediating role of COVID-19 anxiety in the relationship between quality of life and hopelessness in cancer patients. In this study, some subdimensions of quality of life indirectly mediate hopelessness in some subdimensions through COVID-19 anxiety. This result shows that as the quality of life decreases in cancer patients, it may increase hopelessness by acting as a mediator of COVID-19 anxiety. Our findings suggest that cancer patients may experience less hopelessness and improved quality of life when COVID-19 anxiety is reduced during treatment and care processes.

It is thought that spiritual well-being and quality of life have an influence on the hopelessness of cancer patients, and this influence will be represented in the output variables through some variables. Based on this assumption, the mediating role of COVID-19 anxiety in the effect of spiritual well-being and quality of life on hopelessness in cancer patients was investigated. The findings obtained in this context show that COVID-19 anxiety has a partial mediating role in the effect of both spiritual well-being and quality of life on hopelessness. Spiritual well-being and quality of life can reduce the level of hopelessness by strengthening the coping mechanisms of cancer patients. It can strengthen the mental resistance of patients by increasing positive thoughts. On the contrary, patients with low spiritual well-being and quality of life have a higher level of hopelessness. Cancer is a life-threatening disease that causes anxiety. COVID-19 anxiety may increase due to the negativities that may be experienced in care and treatment during the pandemic. Therefore, when spiritual well-being and quality of life decrease during the pandemic, hopelessness will increase through the anxiety of COVID-19.

### Implications for nursing

The contribution of this study lies in highlighting the mediating role of COVID-19 anxiety on the effects of spiritual well-being and quality of life in cancer patients on their hopelessness, which is an important output variable. Second, no study investigating the mediating role of COVID-19 anxiety in the relationship between these 2 independent variables and hopelessness has been found. The finding that hopelessness increases as quality of life and spiritual well-being declines in cancer patients is the first scientific evidence for the mediating role of COVID-19 anxiety. It can be said that the contribution of this study to the practice is that quality of life, spiritual well-being, and COVID-19 anxiety are important variables in reducing the hopelessness of cancer patients, and if these 3 variables are included in nursing care, an important contribution will be made to increasing hope. It can be said that these variables will contribute significantly to the treatment process and the quality of care for cancer patients. For this reason, it is shown that by enhancing quality of life and spiritual well-being through interventions and reducing COVID-19 anxiety, hopelessness among cancer patients can be alleviated. This study shows that spiritual well-being may be important in reducing hopelessness and anxiety and improving quality of life for cancer patients in situations that may occur in the future, such as the COVID-19 pandemic.

### Limitations

This study has several limitations that bear upon the generalizability and interpretation of the fndings and should be addressed. The first cross-sections are the use of the cross-sectional pattern. Therefore, examining the limitations between variables and determining causality becomes very difficult. This study data was collected from university hospital located in western Türkiye. In addition, the spiritual well-being, hopelessness, and quality of life of cancer patients participating in this study were evaluated. Significant differences can be seen among cancer patients in their perception of these concepts. Therefore, it may not be possible to generalize the results of the study to all cancer patients. Finally, it was assumed that the patients gave sincere and precise responses to the measurement tools, but this may not have been the case. Regarding future research, further studies are recommended to overcome some of these limitations in order to gain a clearer understanding of the relationships among quality of life, spiritual well-being, anxiety, and hopelessness.

## Conclusion

It can be concluded that spiritual well-being, quality of life, and the relationship between COVID-19 anxiety and hopelessness are important factors to consider in the comprehensive healthcare of cancer patients. This information can contribute to nurses’ awareness and allow them to adopt a more supportive attitude. Additionally, it is recommended to incorporate practices that increase hope and support spiritual development at every stage of care. During the COVID-19 pandemic, it was expected that minimizing cancer patients’ anxiety and hopelessness would have a significant impact on their care and treatment process. In this context, such findings could serve as a guide for possible similar situations that may have a significant impact on cancer patients in the future. Considering such factors, and taking appropriate action is likely to increase the quality of life and satisfaction of cancer patients.

## Data Availability

The datasets used and analyzed in the current study are available from the corresponding author on reasonable request.

## References

[ref1] Adzrago D, Sulley S, Tagoe I, et al. (2022) Assessment of anxiety/depression among cancer patients before and during the COVID-19 pandemic. *Psycho-Oncology* 31(10), 1681–1691. doi:10.1002/pon.6026 36029183 PMC9762178

[ref2] Akkuzu H, Yumuşak FN, Karaman G, et al. (2020) Koronavirüs Kaygı Ölçeği’nin Türkçe güvenirlik ve geçerlik çalışmaı. *Kibris Tu¨rk Psikiyatri Ve Psikoloji Dergisi* 2(2), 63–67. doi:10.35365/ctjpp.20.2.09

[ref3] Aktürk Ü, Erci B and Araz M (2017) Functional evaluation of treatment of chronic disease: Validity and reliability of the Turkish version of the Spiritual Well-Being Scale. *Palliative and Supportive Care* 15(6), 684–692. doi:10.1017/S1478951517000013 28183363

[ref4] Algahtani FD, Hassan SU, Alsaif B, et al. (2021) Assessment of the quality of life during COVID-19 pandemic: A cross-sectional survey from the Kingdom of Saudi Arabia. *International Journal of Environmental Research & Public Health* 18(3), 847. doi:10.3390/ijerph18030847 PMC7863741 33498152

[ref5] Al-Natour A, Al Momani SM and Qandil AMA (2017) The relationship between spirituality and quality of life of Jordanian women diagnosed with breast cancer. *Journal of Religion and Health* 56(6), 2096–2108. doi:10.1007/s10943-017-0370-8 28168582

[ref6] Andrei F, Mancini G, Agostini F, et al. (2022) Quality of life and job loss during the COVID-19 pandemic: Mediation by hopelessness and moderation by trait emotional intelligence. *International Journal of Environmental Research & Public Health* 19(5), 2756. doi:10.3390/ijerph19052756 PMC8910407 35270449

[ref7] Arndt V, Koch-Gallenkamp L, Jansen L, et al. (2017) Quality of life in long-term and very long-term cancer survivors versus population controls in Germany. *Acta Oncologica (Stockholm, Sweden)* 56(2), 190–197. doi:10.1080/0284186X.2016.1266089 28055266

[ref8] Beck AT, Weissman A, Lester D, et al. (1974) The measurement of pessimism: The hopelessness scale. *Journal of Consulting and Clinical Psychology* 42(6), 861–865. doi:10.1037/h0037562 4436473

[ref9] Bray F, Laversanne M, Sung H, et al. (2024) Global cancer statistics 2022: GLOBOCAN estimates of incidence and mortality worldwide for 36 cancers in 185 countries. *CA: A Cancer Journal for Clinicians* 74(3), 229–263. doi:10.3322/caac.21834 38572751

[ref10] Büyükbayram Z, Aksoy M and Şayan G (2022) Analysis of the spiritual orientations and the hopelessness levels of the patients diagnosed with COVID-19: A cross-sectional study. *Florence Nightingale Journal of Nursing* 30(2), 182–189. doi:10.54614/FNJN.2022.21068 35699636 PMC9449743

[ref11] Cankurtaran ES, Ozalp E, Soygur H, et al. (2008) Understanding the reliability and validity of the EORTC QLQ-C30 in Turkish cancer patients. *European Journal of Cancer Care* 17(1), 98–104. doi:10.1111/j.1365-2354.2007.00827.x 18181898

[ref12] Choi EPH, Hui BPH, Wan EYF, et al. (2021) COVID-19 and health-related quality of life: A community-based online survey in Hong Kong. *International Journal of Environmental Research & Public Health* 18(6), 3228. doi:10.3390/ijerph18063228 PMC8003940 33804725

[ref13] Ciążyńska M, Pabianek M, Szczepaniak K, et al. (2020) Quality of life of cancer patients during coronavirus disease (COVID-19) pandemic. *Psycho-Oncology* 29(9), 1377–1379. doi:10.1002/pon.5434 32779778 PMC7323427

[ref14] Durak A and Palabıyıkoğlu R (1994) Beck Hopelessness Scale validity study. *Crisis Magazine* 2(2), 311–319.

[ref15] Durmuş M, Çiftci N, Gerçek A, et al. (2022) The effect of COVID−19 crisis on hopelessness, loneliness and spiritual well-being of patients with type 1 and type 2 diabetes in Turkey. *Journal of Religion and Health* 61(2), 1703–1718. doi:10.1007/s10943-021-01496-z 35025008 PMC8756405

[ref16] Durmuş M and Öztürk Z (2022) The effect of COVID-19 outbreak on older adults’ hopelessness, loneliness and spiritual well-being in Turkey. *Journal of Religion and Health* 61(1), 851–865. doi:10.1007/s10943-021-01494-1 34997453 PMC8740876

[ref17] Erşen O, Gojayev A, Mercan Ü, et al. (2020) Evaluation of cancer patients’ awareness and fear of COVID-19 and access to health services during the pandemic process. *Turkiye Klinikleri Journal of Medical Sciences* 40(4), 399–405. doi:10.5336/medsci.2020-79092

[ref18] Eskelinen M, Korhonen R, Selander T, et al. (2014) The self-rating score (SRS) versus the examiner rating score (ERS) in measuring helplessness in healthy individuals and in patients with benign breast disease and breast cancer: A prospective case-control study in Finland. *Anticancer Research* 34(10), 5677–5682. 25275073

[ref19] Fardin MA (2020) COVID-19 epidemic and spirituality: A review of the benefits of religion in times of crisis. *Jundishapur Journal of Chronic Disease Care* 9(2), 1–4. doi:10.5812/jjcdc.104260

[ref20] Fayers PM, Aaronson NK, Bjordal K, et al. (2001) *The EORTC QLQ-C30 Scoring Manual, European Organisation for Research and Treatment of Cancer*, 3rd edn. Brussels: European Organisation for Research and Treatment of Cancer.

[ref21] Firkins J, Hansen L, Driessnack M, et al. (2020) Quality of life in “chronic” cancer survivors: A meta-analysis. *Journal of Cancer Survivorship: Research and Practice* 14(4), 504–517. doi:10.1007/s11764-020-00869-9 32162194

[ref22] Flyum IR, Mahic S, Grov EK, et al (2021) Health-related quality of life in patients with colorectal cancer in the palliative phase: A systematic review and meta-analysis. *BMC Palliative Care* 20(1), 1–18. doi:10.1186/s12904-021-00837-9 34530833 PMC8447559

[ref23] Gheihman G, Zimmermann C, Deckert A, et al. (2016) Depression and hopelessness in patients with acute leukemia: The psychological impact of an acute and life-threatening disorder. *Psycho-Oncology* 25(8), 979–989. doi:10.1002/pon.3940 26383625

[ref24] Grajek M, Krupa-Kotara K, Rozmiarek M, et al. (2022) The level of COVID-19 anxiety among oncology patients in Poland. *International Journal of Environmental Research & Public Health* 19(18), 11418. doi:10.3390/ijerph191811418 PMC9517625 36141692

[ref25] Hamilton JB, Best NC, Barney TA, et al. (2022) Using spirituality to cope with COVID-19: The experiences of African American breast cancer survivors. *Journal of Cancer Education* 37(5), 1422–1428. doi:10.1007/s13187-021-01974-8 33595772 PMC7886845

[ref26] Hayes AF (2018) *Introduction to Mediation, Moderation, and Conditional Process Analysis*. New York: The Guilford Press.

[ref27] İzci F, Sarsanov D, Erdogan Zİ, et al. (2018) Impact of personality traits, anxiety, depression and hopelessness levels on quality of life in the patients with breast cancer. *European Journal of Breast Health* 14(2), 105–111. doi:10.5152/ejbh.2018.3724 29774319 PMC5939973

[ref28] Jimenez-Fonseca P, Lorenzo-Seva U, Ferrando PJ, et al. (2018) The mediating role of spirituality (meaning, peace, faith) between psychological distress and mental adjustment in cancer patients. *Supportive Care in Cancer* 26(5), 1411–1418. doi:10.1007/s00520-017-3969-0 29143135

[ref29] Kasapoğlu F (2022) The relationship among spirituality, self-efficacy, COVID-19 anxiety, and hopelessness during the COVID-19 process in Turkey: A path analysis. *Journal of Religion and Health* 61(1), 767–785. doi:10.1007/s10943-021-01472-7 34988842 PMC8731196

[ref30] Khan S, Siddique R, Li H, et al. (2020) Impact of coronavirus outbreak on psychological health. *Journal of Global Health* 10(1), 010331. doi:10.7189/jogh.10.010331 PMC7180007 32355556

[ref31] Kirca N, Adibelli D, Toptas T, et al. (2022) The relationship between spiritual well-being, hope and depression in gynecologic oncology patients. *Health Care for Women International* 1–22. Advance online publication. doi:10.1080/07399332.2021.1995387 35072585

[ref32] Korkut S (2022) Research of the coronavirus anxiety, post-traumatic stress, generalized anxiety disorder, quality of life, and stress coping styles in COVID-19 survivors. *Psychological Reports* 125(6), 3069–3083. doi:10.1007/s10943-017-0370-8 36134735 PMC9500426

[ref33] Lee SA (2020) Coronavirus Anxiety Scale: A brief mental health screener for COVID-19 related anxiety. *Death Studies* 44(7), 393–401. doi:10.1080/07481187.2020.1748481 32299304

[ref34] Luo D, Mei B, Wang P, et al (2024) Prevalence and risk factors for persistent symptoms after COVID-19: A systematic review and meta-analysis. *Clinical Microbiology and Infection: The Official Publication of the European Society of Clinical Microbiology and Infectious Diseases* 30(3), 328–335. doi:10.1016/j.cmi.2023.10.016 37866679

[ref35] Madani H, Pourmemari M, Moghimi M, et al. (2018) Hopelessness, perceived social support and their relationship in Iranian patients with cancer. *Asia-Pacific Journal of Oncology Nursing* 5(3), 314–319. doi:10.4103/apjon.apjon_5_18 29963594 PMC5996580

[ref36] Méndez R, Balanzá-Martínez V, Luperdi SC, et al. (2021) Short-term neuropsychiatric outcomes and quality of life in COVID-19 survivors. *Journal of Internal Medicine* 290(3). doi:10.1111/joim.13262 PMC8013333 33533521

[ref37] Mert S, Peker Karatoprak A, Demirhan Y, et al. (2022) COVID-19, anxiety, and hopelessness: Quality of life among healthcare workers in Turkey. *Evaluation and the Health Professions* 45(1), 97–107. doi:10.1177/01632787211067530 34937430

[ref38] Nayeri ND, Bakhshi F, Khosravi A, et al. (2020) The effect of complementary and alternative medicines on quality of life in patients with breast cancer: A systematic review. *Indian Journal of Palliative Care* 26(1), 95–104. doi:10.4103/IJPC.IJPC_183_19 32132792 PMC7017686

[ref39] Nierop-van Baalen C, Grypdonck M, van Hecke A, et al. (2020) Associated factors of hope in cancer patients during treatment: A systematic literature review. *Journal of Advanced Nursing* 76(7), 1520–1537. doi:10.1111/jan.14344 32133663

[ref40] Pahlevan Sharif S, Lehto RH, Amiri M, et al. (2020) Spirituality and quality of life in women with breast cancer: The role of hope and educational attainment. *Palliative and Supportive Care* 19(1), 55–61. doi:10.1017/S1478951520000383 32580795

[ref41] Pappa S, Ntella V, Giannakas T, et al. (2020) Prevalence of depression, anxiety, and insomnia among healthcare workers during the COVID-19 pandemic: A systematic review and meta-analysis. *Brain, Behavior, and Immunity* 88, 901–907. doi:10.1016/j.bbi.2020.05.026 32437915 PMC7206431

[ref42] Peterman AH, Fitchett G, Brady MJ, et al. (2002) Measuring spiritual well-being in people with cancer: The functional assessment of chronic illness therapy-Spiritual Well-being Scale (FACIT-Sp). *Annals of Behavioral Medicine: A Publication of the Society of Behavioral Medicine* 24(1), 49–58. doi:10.1207/S15324796ABM2401_06 12008794

[ref43] Ravindran OS, Shankar A and Murthy T (2019) A comparative study on perceived stress, coping, quality of life, and hopelessness between cancer patients and survivors. *Indian Journal of Palliative Care* 25(3), 414–420. doi:10.4103/IJPC.IJPC_1_19 31413458 PMC6659528

[ref44] Rawas H, De Beer J, Alturki O, et al. (2024) Hopelessness and social support among cancer patients in Saudi Arabia. *Asian Pacific Journal of Cancer Prevention: APJCP* 25(4), 1363–1370. doi:10.31557/APJCP.2024.25.4.1363 38679998 PMC11162714

[ref45] Saarelainen SM (2019) Landscapes of hope and despair: Stories of the future of emerging adults in cancer remission. *Journal of Pastoral Theology* 29(2), 67–84. doi:10.1080/10649867.2019.1632403

[ref46] Saricali M, Satici SA, Satici B, et al. (2022) Fear of COVID-19, mindfulness, humor, and hopelessness: A multiple mediation analysis. *International Journal of Mental Health and Addiction* 20(4), 2151–2164. doi:10.1007/s11469-020-00419-5 33230394 PMC7676415

[ref47] Sekaran U (2019) *Research Methods for Business*. Canada: John Wiley ve Sons.

[ref48] Thong MSY, Koch-Gallenkamp L, Jansen L, et al. (2019) Age-specific health-related quality of life in long-term and very long-term colorectal cancer survivors versus population controls - a population-based study. *Acta Oncologica (Stockholm, Sweden)* 58(5), 801–810. doi:10.1080/0284186X.2018.1557340 30736716

[ref49] Vincensi BB (2019) Interconnections: Spirituality, spiritual care, and patient-centered care. *Asia-Pacific Journal of Oncology Nursing* 6(2), 104–110. doi:10.4103/apjon.apjon_48_18 30931353 PMC6371669

[ref50] Wang Y and Feng W (2022) Cancer-related psychosocial challenges. *General Psychiatry* 35(5), e100871. doi:10.1136/gpsych-2022-100871 PMC9540834 36311374

